# microRNA-503 contribute to pancreatic beta cell dysfunction by targeting the mTOR pathway in gestational diabetes mellitus

**DOI:** 10.17179/excli2017-738

**Published:** 2017-10-27

**Authors:** Ke Xu, Dezhi Bian, Lanxiang Hao, Fei Huang, Min Xu, Jie Qin, Yanmei Liu

**Affiliations:** 1 Department of Endocrinology, Yancheng First City Hospital of Jiangsu Province, Yancheng, China; 2 Department of Obstetrics and Gynecology, Yancheng First City Hospital of Jiangsu Province, Yancheng, China; 3 Department of Pediatrics, Yancheng First City Hospital of Jiangsu Province, Yancheng, China

**Keywords:** gestational diabetes mellitus, microRNA-503, pancreatic beta-cells, mTOR

## Abstract

Loss of pancreatic β cells is involved in pathogenesis of gestational diabetes mellitus (GDM). Recently, several studies have elucidated the connection between microRNAs (miRNAs) and diabetes mellitus (DM), but the role of miRNAs in GDM remains unclear. The aim of this study was to evaluate the potential functions of miRNAs in GDM and to investigate the underlying mechanisms of action. First, we explored the expression profile of miRNAs in placenta tissue from GDM patients using microarray. Validation analysis was performed in peripheral blood specimens using quantitative reverse transcription PCR (qRT-PCR). Then the role and regulating mechanism of miR-503 in weaken the function of pancreatic β cell was investigated. We found that miR-503 was markedly upregulated in placenta tissue from GDM patients, as elevated in peripheral blood specimens, and the high level was positively correlated to blood glucose concentration. Knockdown of miR-503 enhanced insulin secretion of pancreatic β-cells, promoted cell proliferation and protected cells from apoptosis, whereas overexpression of miR-503 showed the opposite effects. Furthermore, mammalian target of rapamycin (mTOR) was identified as a direct target of miR-503 and mTOR silencing could reverse the improving effects of miR-503 knockdown on insulin secretion and pancreatic β-cells proliferation. High expression of miR-503 in peripheral blood may be acted as a diagnosis biomarker of GDM. MiR-503 regulated functions of pancreatic β-cells by targeting the mTOR pathway, suggesting that targeting miR-503/mTOR axis could serve as a novel therapeutic target for GDM.

## Introduction

Gestational diabetes mellitus (GDM) is one of the most common pathological conditions that can develop during pregnancy and present the leading cause of maternal and fetal morbidity and mortality (Roberts et al., 2003[26]). Although GDM usually disappears after delivery, women with poorly managed GDM are at a greater risk of experiencing adverse pregnancy outcomes, and both mother and child are at increased risk of developing type 2 diabetes (T2D) later in life (Kim et al., 2002[15]; Fetita et al., 2006[11]; Bellamy et al., 2009[3]). Therefore, it is of great importance to understand the mechanisms leading to the development of GDM.

Several studies have shown that the underlying cause of GDM is probably related to an exacerbation of the β cell dysfunction which cannot properly secrete insulin in response to hyperglycemia (Buchanan, 2001[5]; Wang et al., 2007[29]). Normal pregnancy is usually accompanied by progressive insulin resistance (IR) (Catalano et al., 1999[8]). Once IR occurs in peripheral tissues, the pancreas first increases insulin release by improving the function of β cells or increasing their number in order to maintain maternal euglycemia. Recent studies found that lipotoxicity-induced β cell dysfunction was a leading cause of the development of GDM (MacDonald et al., 1992[19]; Reaven, 1993[24]). Retnakaran et al. showed that the furan fatty acid metabolite, 3-carboxy-4-methyl-5-propyl-2-furanpropanoic acid (CMPF) caused β cell dysfunction at concentrations found in plasma from GDM and T2D patients through impairment of mitochondrial function and inhibition of insulin biosynthesis (Retnakaran et al., 2016[25]). However, the molecular mechanism of pancreatic β cells dysfunction in the process of GDM remains unclear.

MicroRNAs (miRNAs) are a class of small non-coding RNAs and about 22 nucleotides in length, which regulate gene function by targeting mRNAs for translational repression or degradation (Bartel, 2004[2]). Increasing evidence indicates that miRNAs are involved in the pathogenesis of diabetes and that a number of miRNAs have been reported to be differently expressed in GDM, such as miR-132, miR-181a, miR-29c and so on (Zhao et al., 2011[32]; Collares et al., 2013[10]). Specifically, miRNAs are required for pancreatic β cells function and the regulation of glucose stimulated insulin secretion (Poy et al., 2004[22]; Plaisance et al., 2006[21]; Lynn et al., 2007[18]). Therefore, more studies need to be performed to identify more novel miRNAs in GDM.

In this study, we focused the expression level of miR-503 in GDM and studied its role in the functions of pancreatic β cells. Our results showed that inhibition of miR-503 could enhance insulin secretion and cell viability of pancreatic β cells, while suppress pancreatic β cells apoptosis by directly targeting mTOR. These data suggest that targeting miR-503/mTOR pathway might be a novel therapeutic target for GDM.

## Materials and Methods

### Subjects and sample collection

All the subjects were recruited from the Yancheng City Hospital of Jiangsu Province (Yancheng, China) in 2015-2016. Written informed consent was obtained prior to inclusion and the present study was approved by the Ethics Committee of the Yancheng City Hospital of Jiangsu Province (Yancheng, China).

Placentas tissues were obtained from 3 women with normal pregnancies and 3 women with GDM and processed immediately. Peripheral blood samples (2.5 ml) from patients with GDM (n=25) and normal subjects (n=25) were collected from the the Yancheng City Hospital of Jiangsu Province from 2014 to 2015. Patients with serious liver or kidney diseases, malignancy and acute heart failure were excluded. All samples were flash-frozen in liquid nitrogen, and stored at -80 °C until further molecular analysis.

### microRNA expression profiling

Total RNA was extracted from placenta tissues using the miRNeasy mini kit (Qiagen, West Sussex, UK). Samples were labeled with a label kit (miRCURY™ Hy3™/Hy5™ Power labeling kit and hybridized to the miRCURY LNA™ Array (v.18.0) (Agilent Technologies). The chips were scanned with the Axon GenePix 4000B Microarray Scanner. The procedure and images process method as described previously (Xu et al., 2017[31]). The miRNA expressions of all differentially expressed samples were clearly displayed by a hierarchical clustering heat map.

### Quantitative reverse transcription PCR (qRT-PCR)

Total RNA was extracted from peripheral blood, placenta tissues and cells using the PAXgene Blood miRNA kit (PreAnalytix), the miRNeasy mini kit (Qiagen, West Sussex, UK) and TRIzol reagent (Invitrogen) according to the manufacturer's instructions. Then the RNA was reversely transcribed to cDNA. The specific primer was designed with Primer 5.0 software and synthesized by the Shanghai Sangon Biotech Company. Specific gene levels were detected with a real-time PCR reaction system and reaction parameters.

### Cell culture

The INS-1 cells were maintained in Dulbecco's modified Eagle's medium containing 11 mol/L glucose supplemented with 10 % heat-inactivated fetal bovine serum (Gibco), 100 units/ml penicillin, 100 μg/ml streptomycin and 50 µM β-mercaptoethanol at 37 °C in a humidified atmosphere of 5 % CO_2_. Human 293T embryonic kidney cells were purchased from the American Type Culture Collection (Manassas, VA, USA) and maintained in Dulbecco's modified Eagle's medium (Invitrogen) containing 10 % fetal bovine serum (Invitrogen) and 1/100 streptomycin-penicillin mix (Sigma) and incubated at 37 °C with 5 % CO_2_.

### Cell transfection

MiR-503 mimics, miR-503 inhibitor and controls were purchased from Shanghai GenePharma (Shanghai, China). The mTOR siRNA and NC siRNA were purchased from Santa Cruz Biotechnology (Santa Cruz, CA, USA). INS-1 cells were seeded at 4×10^5^ cells/well in 6-well plates, and cultured in antibiotic-free DMEM at 37 °C and 5 % CO_2_. When the cell density reached 30-50 %, cell transfection was performed using Lipofectamine 2000 (Invitrogen, Carlsbad, CA, USA) according to the manufacturer's instructions. 48 hours after transfection, cells were collected for further experiments.

### Detection of glucose-stimulated insulin secretion

The cells were seeded in a 96-well plate and cultured for 24 h. The cells were then treated with basal glucose (5.5 mM) or stimulatory glucose (22 mM) for 1 h. Subsequently, the insulin level was measured by enzyme-linked immunosorbent assay (ELISA). Total insulin content was measured after sonication of cells in acid ethanol (2% H_2_SO_4_), followed by 3 freeze/thaw cycles, and then centrifuged for 5 min at 10,000 × g. The supernatant was used to measure the insulin level by ELISA as described above.

### Cell viability assay

3-(4,5-dimethylthiazol-2-yl)-2,5-diphenyltetrazolium bromide (MTT) assay was performed to detect the effect of miR-503 on cell viability. Briefly, cells were seeded in 96-well plates at a density of 2 × 10^3^ cells/well in 100 μL growth medium. After transfection, cells were treated with 20 μl MTT solution (5 mg/ml) for 4 h. Then, the absorbance at a wavelength of 490 nm was detected by a microplate reader (Bio-Tek Instruments, Winooski, VT, USA) according to the manufacturer's instructions.

### Apoptosis assay

Flow cytometry was applied to detect the effect of miR-503 on cell apoptosis. Cultured or transfected cells were collected, washed with PBS and re-suspended in 500 μl of binding buffer. After the density of the cells was adjusted to 5 × 10^5^/ml, cells were incubated with 5 μl Annexin V and 5 μl propidium iodide (PI) (BD Biosciences) at room temperature in the dark for 15 min. Apoptotic keratinocytes were quantified by flow cytometry (BD Biosciences).

### Luciferase reporter assay

A cDNA fragment of the mTOR 3´-UTR mRNA containing the seed sequence of the miR-503-binding site or a mutated binding site was cloned into the pmirGLO dual-luciferase vector (Promega, Madison, WI, USA). The constructed dual-luciferase vector was co-transfected with miR-503 mimics, miR-503 inhibitor or NC into HEK293T cells. After 48 h of incubation, cells were collected for application in the Dual-Luciferase Reporter System (Promega, Madison, WI) following the manufacturer's recommendations. All of the dual-luciferase reporter assays were done in triplicate within each experiment, and three independent experiments were conducted.

### Enzyme-linked immunosorbent assay (ELISA)

The measurement of mTOR ELISA was performed according to the manufacturer's instructions (Cell Signaling Technology, USA). All assays were done in triplicate within each experiment, and three independent experiments were conducted. Protein levels were calculated as pg/mg of total proteins.

### Western blot

Total proteins from transfected cells were extracted using RIPA lysis buffer containing proteinase inhibitor (Sigma, USA). Protein samples (40 μg) were analyzed by 8 % SDS-PAGE gel and transferred onto the PVDF membrane (Millipore). After being blocked in 10 % nonfat dried milk for 2 h, the blots were incubated with primary antibodies against mTOR (1:1000; Abcam, Cambridge, UK) at 4 °C overnight. After washing three times with TBST, the blots were incubated with secondary antibody at room temperature for 1 h. After washing three times with TBST, the protein bands were visualized by enhanced chemiluminescence detection reagents (Applygen Technologies Inc., Beijing, China). Relative band intensities were determined by densitometry using Scion image software (version 4.0). Control antibodies were anti-β-actin (1:2000; Santa Cruz, CA, USA).

### Statistical analysis

Statistical analysis was performed using GraphPad Prism 5.0 (GraphPad Software, Inc., San Diego, CA, USA). Differences were analyzed with the Student's t-test between two groups or with one-way ANOVA among four groups. Correlation analyses were made with Spearman's correlation analysis test. A p-value of less than 0.05 was considered statistically significant.

## Results

### miR-503 is upregulated in GDM and its expression is positively correlated with blood glucose concentration

**Figure 1:**
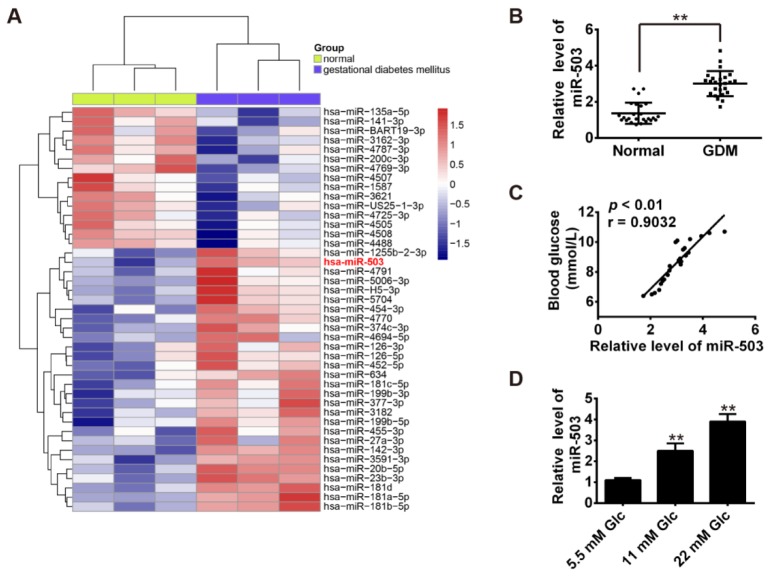
miR-503 is upregulated in GDM and its expression is positively correlated with blood glucose concentration. A. Heatmap of normalized expression levels of miRNAs in placenta tissues from 3 pairs of GDM pregnancies and healthy pregnancies. B. qRT-PCR was performed to determine the expression levels of miR-503 in peripheral blood samples from 25 pairs of GDM pregnancies and healthy pregnancies. **p < 0.01 vs. Normal group. C. Correlation between miR-503 level and blood glucose determined by Spearman correlation analysis (r = 0.9032, *p* < 0.01). D. qRT-PCR was performed to determine the expression levels of miR-503 in INS-1 cells maintained for 4 days with different glucose concentrations (5.5 mM, 11 mM and 22 mM). 5.5 mM glucose acts as an internal control. Data represent the mean ± SD of three independent experiments. **p < 0.01 compared with 5.5mM Glc group.

To elucidate the expression of miRNAs associated with GDM, we performed a miRNA microarray on placenta tissues from 3 pairs of GDM pregnancies and healthy pregnancies. Our results revealed that 28 miRNAs were upregulated and 15 miRNAs were downregulated in GDM group when compared with the normal group (Figure 1A(Fig. 1)). Among these aberrantly expressed miRNAs, miR-503 was one of miRNAs being most significantly upregulated. Based on several studies it has been reported that the expression of miR-503 was increased in diabetes mellitus (Caporali et al., 2011[7], 2015[6]; Zhu et al., 2016[34]), we tested if miR-503 was also involved in the pathogenetic procession of GDM. Consistent with the array results, real-time RT-PCR further validated the finding that miR-503 was significantly up-regulated in peripheral blood samples (Figure 1B(Fig. 1)). Furthermore, a positive correlation between the expression level of miR-503 and blood glucose concentration was observed in patients with GDM (R =0.9032, p < 0.01, Figure 1C(Fig. 1)). Our data suggest that high expression of miR-503 may be a novel diagnostic marker for GDM treatment. 

### Knockdown of miR-503 enhanced the functions of pancreatic β cell

To explore the roles of miR-503 in GDM, the INS-1 cell line was applied as it is reported to share many characteristics of pancreatic β cells (including insulin secretion) in response to glucose stimulation (Li et al., 2016[17]). First, we analyzed the expression of miR-503 in INS-1 cells maintained for 4 days with different glucose concentrations. As shown in Figure 1D(Fig. 1), miR-503 induction was dose-dependent at glucose concentrations of 5.5-22 mM.

**Figure 2:**
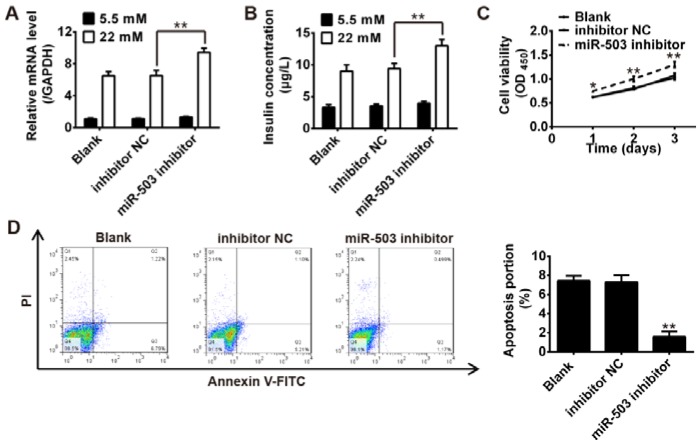
Knockdown of miR-503 enhanced the functions of pancreatic β cell. miR-503 inhibitor was transfected into INS-1 cells pretreated with 5.5 mM glucose or 22 mM high glucose for 24 h. A. Insulin mRNA levels were measured by qRT-PCR. B. Total insulin content was measured by ELISA assay. C. Cell viability was determined by MTT assay. D. Cell apoptosis was detected by flow cytometry. Data represent the mean ± SD of three independent experiments. *p < 0.05, **p < 0.01 compared with inhibitor NC or Blank group.

As the expression level of miR-503 is increased under high glucose, we transfected a miR-503 inhibitor to reduce its expression level. Following transfection, then insulin mRNA levels and total insulin content were measured by qRT-PCR and ELISA assay. The results showed that knockdown of miR-503 markedly increased insulin expression and insulin content in response to glucose stimulation when compared with the control and inhibitor NC groups (Figure 2 A, B(Fig. 2)). We further examined the effect of miR-503 inhibition on the viability and apoptosis of pancreatic β cells. Our data indicated that the inhibition of miR-503 significantly enhanced cell proliferation (Figure 2C(Fig. 2)), while it suppressed the apoptosis of the INS-1 cells (Figure 2D(Fig. 2)), when compared with the control and inhibitor NC groups. These results suggest that miR-503 inhibition enhances the proliferation and insulin secretion, while it suppresses the apoptosis of pancreatic β cells.

### Overexpression of miR-503 suppressed the functions of pancreatic β cell

**Figure 3:**
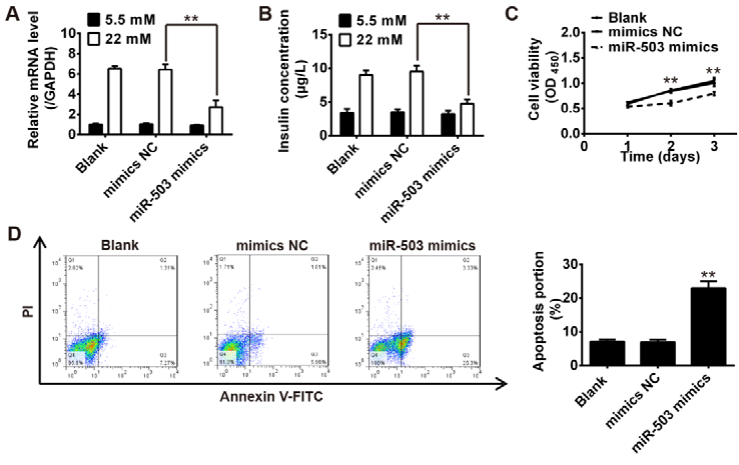
Overexpression of miR-503 suppressed the functions of pancreatic β cell. miR-503 mimics was transfected into INS-1 cells pretreated with 5.5 mM glucose or 22 mM high glucose for 24 h. A. Insulin mRNA levels were measured by qRT-PCR. B. Total insulin content was measured by ELISA assay. C. Cell viability was determined by MTT assay. D. Cell apoptosis was detected by flow cytometry. Data represent the mean ± SD of three independent experiments. **p < 0.01 compared with mimics NC or Blank group.

To further reveal the role of upregulation of miR-503 in pancreatic β cell function, we transfected a miR-503 mimics into INS-1 cells pretreated with 5.5 mM glucose or 22 mM high glucose for 24 h. After 24 h transfection, insulin mRNA levels and total insulin content were measured by qRT-PCR and ELISA assay. The results showed that overexpression of miR-503 markedly decreased insulin expression and insulin content in response to glucose stimulation when compared with the control and mimics NC groups (Figure 3 A, B(Fig. 3)). We also examined the effect of miR-503 mimics on the viability and apoptosis of pancreatic β cells response to glucose stimulation. Our data indicated that the overexpression of miR-503 significantly inhibited cell proliferation (Figure 3C(Fig. 3)), while it promoted the apoptosis of the INS-1 cells (Figure 3D(Fig. 3)), when compared with the control and mimics NC groups. These results suggest that miR-503 mimics inhibits the proliferation and insulin secretion, while it induces the apoptosis of pancreatic β cells.

### mTOR is a direct target of miR-503

**Figure 4:**
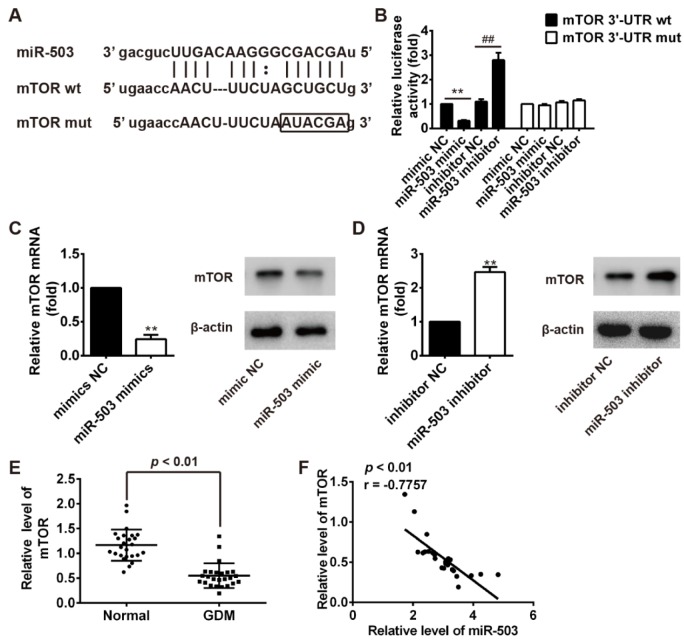
mTOR was a direct target of miR-503. A. The predicted miR-503 binding sites on mTOR. B. Luciferase activity in HEK293T cells co-transfected with miR-503 mimics, miR-503 inhibitor and luciferase reporters containing mTOR wild type or mutant type (MUT) 3´-UTR. Histogram indicates the values of luciferase measured 48 h after transfection. **p < 0.01 vs. mimic NC group; ##*p* < 0.01 vs. inhibitor NC group. C and D. miR-503 mimics, miR-503 inhibitor and controls were transfected into INS-1 cells, then the mRNA and protein levels of mTOR were detected by qRT-PCR and Western Blot. **p < 0.01 vs. mimic NC or inhibitor NC group. E. The expression level of mTOR in peripheral blood samples from 25 pairs of GDM pregnancies and healthy pregnancies was detected by using qRT-PCR. **p < 0.01 vs. Normal group. F. Pearson's correlation analysis of the relationship between miR-503 expression and mTOR expression in peripheral blood samples from 25 pairs of GDM pregnancies (r =0-0.7757, *p* < 0.01).

To assess the role of miR-503 in regulating the functions of pancreatic β cells, bioinformatics tools were used to search for potential targets of miR-503. According to the results of these analyses, we focused on mTOR, an atypical serine/threonine kinase since it has recently been found to be involved in sustaining compensatory insulin secretion of pancreatic β cells in response to metabolic stress (Xie et al., 2017[30]). As suggested in Figure 4A(Fig. 4), the complementary sequence of miR-503 was found in the 3´-UTR of mTOR mRNA. To test whether that miR-503 could directly target 3´-UTR of mTOR, a luciferase reporter assay was conducted. The results showed that overexpression of miR-503 significantly decreased the luciferase activity of mTOR with wt 3´-UTR, whereas miR-503 inhibition increased the luciferase activity of mTOR with wt 3´-UTR (Figure 4B(Fig. 4)). 

Likewise, cells co-transfected with miR-503 mimics, miR-503 inhibitor, and mTOR-mut-3´UTR showed no obvious change in their luciferase activity (Figure 4B(Fig. 4)). We further examined whether miR-503 could modulate the expression of mTOR in INS-1 cells. The results of qRT-PCR and Western blot showed that overexpression of miR-503 significantly reduced the expressions of mTOR at protein and mRNA levels, whereas inhibition of miR-503 promoted the expressions of mTOR (Figure 4C, D(Fig. 4)). In addition, the expression of mTOR mRNA was investigated and it was significantly increased in peripheral blood samples from patients with GDM compared with that in normal subjects (Figure 4E(Fig. 4)). To further confirm whether mTOR expression could be negatively regulated by miR-503 in GDM, the association between the expression of miR-503 and mTOR was investigated in peripheral blood samples from patients with GDM. It was demonstrated that miR-503 expression was negatively correlated with mTOR expression in peripheral blood from GDM patients (r=-0.7757; p < 0.01; Figure 4F(Fig. 4)), indicating that miR-503 regulates the expression of mTOR in GDM.

### Inhibition of miR-503 enhanced the functions of pancreatic β cells by targeting mTOR

**Figure 5:**
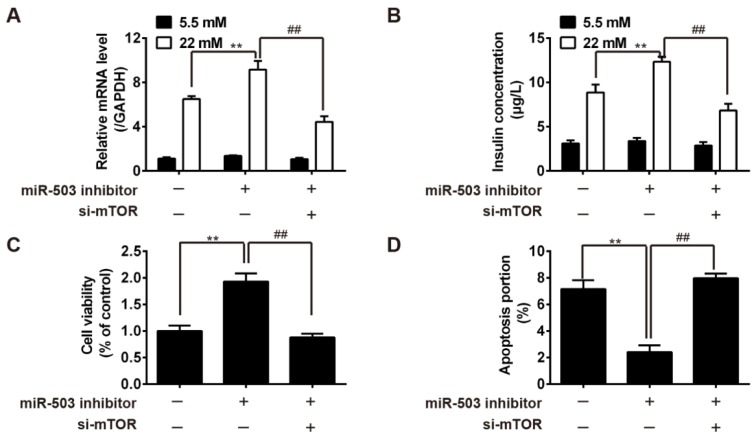
Inhibition of miR-503 enhanced the functions of pancreatic β cells by targeting mTOR. MiR-503 inhibitor and si-mTOR were co-transfected into INS-1 cells pretreated with 5.5 or 22 mM glucose. A. Insulin mRNA levels was measured by qRT-PCR. B. Total insulin content was measured by ELISA assay. C. Cell viability was determined by MTT assay. D. Cell apoptosis was detected by flow cytometry. Data represent the mean ± SD of three independent experiments. **p < 0.01 compared with Blank, ##p < 0.01 vs. miR-503 inhibitor

The transfection of si-mTOR was performed to test if mTOR is involved in the enhanced functions of pancreatic β cells induced by miR-503 inhibitor. qRT-PCR and ELISA results showed that insulin secretion and total insulin content in HG-treated INS-1 were significantly reduced in si-mTOR + miR-503 inhibitor group compared with miR-503 inhibitor group (Figure 5A, B(Fig. 5)). MTT assay revealed that cell viability was also reduced, while flow cytometry assay confirmed that cell apoptosis was promoted in HG-treated INS-1, in comparison with miR-503 inhibitor group (Figure 5C, D(Fig. 5)). Collectively, the interference on mTOR was able to reverse the enhancement of miR-503 inhibition on the functions of pancreatic β cells.

## Discussion

In the present study, we found miR-503 was upregulated in GDM and its expression is positively correlated with blood glucose concentration. Moreover, miR-503 regulated the functions of pancreatic β cells by directly targeting mTOR. Our data suggest that miR-503 may be a potential therapeutic target in GDM.

Several studies have been conducted to evaluate specifically expressed miRNAs in the diverse types of diabetes and diabetes-associated complications (Tang et al., 2008[28], 2009[27]; Hennessy et al., 2010[14]; Caporali et al., 2011[7]). Specifically, miRNAs have been found to be associated with the regulation of insulin production and secretion that is determined by the secretory activity of the β-cell and the total number of β-cells in the pancreas (Bao et al., 2015[1]; Chen et al., 2015[9]; Li et al., 2016[16]). For example, Li et al. showed that miR-19a-3p enhanced the proliferation and insulin secretion, while it inhibited the apoptosis of pancreatic β cells via the inhibition of suppressor of cytokine signaling 3 (SOCS3) (Li et al., 2016[16]). In this study, we investigated the miRNA expression profiles in placenta tissues from GDM pregnancies compared with normal pregnancies and found that miR-503 was upregulated in placenta tissues and peripheral blood samples. We also found that high expression of miR-503 was positively correlated with blood glucose concentration, suggesting that miR-503 may be involved in the procession of GDM.

Recently, miR-503 has been reported to be upregulated in the diverse types of diabetes and diabetes-associated complications (Caporali et al., 2011[7], 2015[6]; Zhu et al., 2016[34]). For example, a recent study from Caporali et al. showed that miR-503 expression was upregulated in the ischemic limb muscles of diabetic mice and patients, and then revealed the significance of miR-503 in diabetes-associated endothelial dysfunction and impaired post-ischemic vascular repair (Caporali et al., 2011[7]). Previous studies demonstrated that several miRNAs were involved in regulation of pancreatic β cells functions, such as miR-101 and miR-30b (Zheng et al., 2015[33]). However, the transcriptional regulatory role of miR-503 in the function of pancreatic β cells has not been investigated yet. In this study, we found that inhibition of miR-503 promoted the proliferation and insulin secretion, while it suppressed the apoptosis of INS-1 under conditions of glucose stimulation. Likewise, overexpression of miR-503 had an opposite effect. These results suggest that miR-503 played an important role in the function of pancreatic β cells. 

mTOR is an evolutionarily conserved protein kinase and forms two functional complexes, termed mTORC1 and mTOR2 complex. Several studies suggest that both mTOR complexes are essential for the regulation of cell growth and survival, and play important roles in maintaining adequate β cell mass (Gu et al.; 2011[12]; Li et al., 2016[16]). Activation of mTOR signaling by growth factors and nutrients promoted pancreatic β-cell replication, expansion of β-cell mass, and improved glucose tolerance (Bernal-Mizrachi et al., 2001[4]; Rachdi et al., 2008[23]; Hamada et al., 2009[13]). In contrast, disruption of mTORC1 signaling decreased β-cell mass and induced hyperglycemia in S6K1-deficient mice (Pende et al., 2000[20]). Recently, Xie et al. found that the mTORC2/PKC pathway sustained compensatory insulin secretion of pancreatic β cells in response to metabolic stress (Xie et al., 2017[30]). In this study, we identified mTOR as a direct target of miR-503. Moreover, miR-503 and mTOR expression were inversely correlated in peripheral blood samples, suggesting that miR-503 negatively regulated the expression of mTOR in GDM. Our results show that suppression of mTOR with siRNA gene silencing reversed the promoting effects of miR-503 inhibition on the proliferation, insulin secretion and the inhibitory effect on the apoptosis of INS-1 under conditions of glucose stimulation. Collectively, these data suggest that miR-503 inhibition exerts its functional role via mTOR pathway in HG-stimulated INS-1 cells.

In conclusion, our finding that miR-503 inhibition promotes the functions of pancreatic β-cell via activating mTOR signaling pathway implicates miR-503 as a novel target for GDM therapy. However, further studies are required to verify our findings *in vivo*. 

## Acknowledgements

This study was supported by the Medical Science and Technology Development Projects of Yancheng City (No.: YK2014006).

## Conflict of interest

We all declare that we have no conflict of interest.
